# Therapeutic Potential of Propolis in Ulcerative Colitis: A Review of Preclinical Evidence

**DOI:** 10.1002/cbdv.71886

**Published:** 2026-10-30

**Authors:** Thayná Fernandes Barros, Gabriel Gomes Vila Nova, Rosane Nassar Meireles Guerra, Flavia Raquel Fernandes do Nascimento, Afonso Gomes Abreu

**Affiliations:** ^1^ Programa De Pós‐graduação em Ciências da Saúde Universidade Federal do Maranhão São Luís Brazil; ^2^ Laboratório De Imunofisiologia Universidade Federal do Maranhão São Luís MA Brazil

**Keywords:** propolis, ulcerative colitis, molecular mechanisms

## Abstract

Propolis is a resinous, aromatic substance collected by worker bees from various plant species. Evidence suggests that propolis is a promising candidate for the treatment of ulcerative colitis due to its antioxidant properties and ability to modulate immune responses and the gut microbiota. This review aimed to analyze studies on the efficacy of propolis and its main active compounds in ulcerative colitis, highlighting mechanisms of action, safety and limitations. A literature search was conducted in the PubMed Central, ScienceDirect, and Scopus databases using the keywords “propolis” AND “colitis”. Original articles presenting in vivo analyses, published in English up to June 2025, were included. Propolis extracts improved clinical and histopathological parameters, particularly by reducing pro‐inflammatory cytokine expression, inflammatory cell infiltration, and restoring epithelial barrier integrity. These effects mainly involve modulation of inflammatory signaling pathways, particularly suppression of NF‐κB activation, resulting in reduced expression of inflammatory mediators, including TNF‐α, IL‐1β, and iNOS. Propolis also reduced TLR4 expression, potentially contributing to the control of the exacerbated immune response characteristic of ulcerative colitis. These findings support the potential of propolis as an adjuvant agent in disease management; however, its clinical efficacy in humans remains to be validated in well‐controlled and standardized studies.

## Graphical abstract

This review compiles preclinical evidence from studies that investigate the therapeutic potential of propolis extracts derived from different botanical origins, as well as their main bioactive constituents, in the context of ulcerative colitis. The observed beneficial effects are primarily attributed to their antioxidant, immunomodulatory, and anti‐inflammatory properties, along with their ability to restore intestinal barrier integrity.

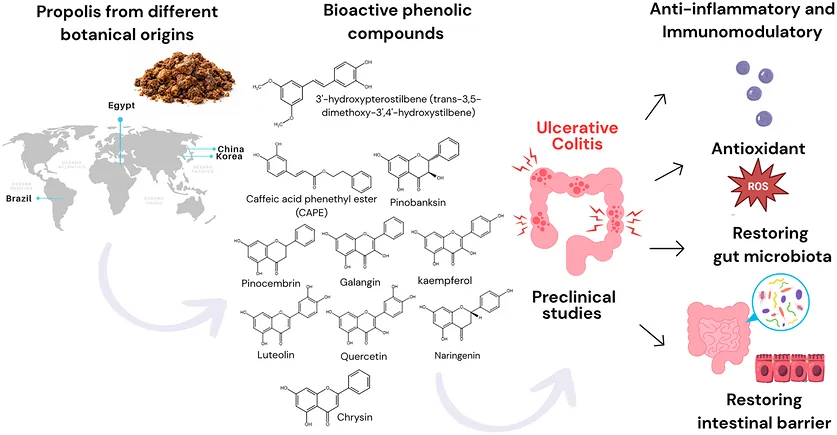

**Abbreviations**
ABTS: 2,2'‐azinobis (3‐ethylbenzothiazoline‐6‐sulfonic acid)
AOM: Azoxymethane
CAT: catalase
COX‐1: cyclooxygenase‐1
COX‐2: cyclooxygenase‐2
DNA: Deoxyribonucleic Acid
DSS: dextran sulfate sodium
FRAP: Ferric Reducing Ability
GSH: glutathione
GSH‐Px: glutathione peroxidase
GST: glutathione‐S‐transferase
IBD: inflammatory bowel disease
ICAM‐1: Intercellular Adhesion Molecule 1
IFNγ: gamma interferon
IL: Interleukin
in vitro: assay under controlled laboratory conditions
in vivo: assay performed in living organisms
iNOS: inducible nitric oxide synthase
JAM‐A: Junctional Adhesion Molecule‐A
LTB_4_
: leukotriene B4
MCP‐1: monocyte chemoattractant protein‐1
MDA: malondialdehyde
MPO: myeloperoxidase
mRNA: messenger RNA
NF‐κB: nuclear factor kappa B
NO: nitric oxide
NP‐SH: nonprotein sulfhydryl
NQO1: quinone oxidoreductase 1
Nrf2: Nuclear factor erythroid 2‐related factor 2
PGE2: prostaglandin
PMC: PubMed Central
PXR: human pregnane X receptor
RNA: Ribonucleic Acid
ROS: reactive oxygen species
SOD: superoxide dismutase
T‐AOC: total antioxidant capacity
TBARS: thiobarbituric acid‐reactive substances
TFF3: Trefoil Factor Family
TGF‐β: transforming growth factor‐β
T‐GSH: total glutathione sulfhydryl
TH1: type 1 T helper cell
TLR4: Toll‐like receptor 4
TNBS: trinitrobenzenesulfonic acid
TNF‐α: tumor necrosis factor‐alpha
TRPV1: transient receptor potential vanilloid 1
UC: ulcerative colitis
ZO‐1: Zonula Occludens‐1

## 1 Introduction

Ulcerative colitis (UC) is a chronic, relapsing‐remitting inflammatory bowel disease (IBD) characterized by inflammation limited to the mucosal layer, beginning in the rectum and extending proximally in a continuous pattern throughout the colon [1, 2]. UC significantly impairs patients’ quality of life and social functioning. Its main clinical manifestations include frequent bowel movements, the presence of mucus and pus, bloody diarrhea, abdominal pain and discomfort, urgency, weight loss, and tenesmus [3, 4].

Globally, UC is estimated to affect approximately 5 million individuals. Its prevalence varies considerably across regions, being higher in Northern Europe and North America (> 198 per 100 000 inhabitants) than in Asia (20–101 per 100 000 inhabitants). Notably, incidence rates are increasing worldwide, particularly in rapidly industrializing regions such as Asia, Africa, and South America [5].

Epidemiological data on IBD in Brazil remain limited. However, a national population‐based study reported an increase in UC incidence from 5.7 to 6.9 per 100 000 inhabitants per year over the past decade [6]. Given the morbidity and mortality associated with UC, the economic burden is substantial. Annual direct and indirect costs are estimated to range from €12.5 to €29.1 billion in Europe and from US$8.1 to US$14.9 billion in the United States [7, 8].

The etiology of UC remains incompletely understood; however, it is widely considered a multifactorial disorder involving interactions among gut microbiota, mucosal immune responses, genetic predisposition, and environmental factors (e.g., medications, diet, and smoking). UC has been proposed to involve an exaggerated immune inflammatory response, potentially triggered by dysbiosis and microbial imbalance [9]. Certain clinical and demographic factors have been associated with distinct disease phenotypes and poorer prognosis. UC is most commonly diagnosed in late adolescence or early adulthood, although it can occur at any age, with similar prevalence in men and women [1, 10].

Current treatment strategies for UC include aminosalicylates, corticosteroids, immunosuppressants, monoclonal antibodies, biological agents, stem cell therapy, fecal microbiota transplantation, helminth therapy, and surgery [11, 12, 13]. However, these therapies may be associated with significant limitations, including adverse effects, drug tolerance, and high recurrence rates. In some cases, treatment failure may exacerbate inflammation and intestinal damage [14, 15]. For instance, aminosalicylates are commonly used in mild to moderate disease, but long‐term use may be associated with side effects, and poor adherence can increase recurrence rates. Likewise, prolonged corticosteroid use has been linked to an increased risk of mortality, particularly in older patients, who may also exhibit greater dependence on these agents [15]. These limitations highlight the need for alternative therapeutic approaches with improved safety profiles and lower toxicity.

In this context, natural products such as propolis have attracted attention as potential therapeutic agents for UC. Propolis is a resinous substance produced by bees that exhibits a wide range of phytochemical and biological properties and has been investigated for various therapeutic applications [16]. Its constituents demonstrate notable anti‐inflammatory and immunomodulatory effects, which may contribute to the regulation of immune responses [17, 18].

Accordingly, this review focuses on the properties of propolis and its therapeutic potential in the treatment of UC. Specifically, it aims to describe the pharmacological properties of propolis, with emphasis on its anti‐inflammatory, antioxidant, and immunomodulatory effects. Furthermore, it evaluates the efficacy of propolis and its active compounds in UC, highlighting the underlying mechanisms of action, as well as considerations related to safety and limitations of its use.

## 2 Methodology

This review was structured as a narrative review rather than a systematic review, since its objective was to synthesize the available preclinical evidence on the properties of propolis and its therapeutic potential for the treatment of ulcerative colitis. The identification, selection, and organization of the studies were conducted in accordance with the PRISMA (Preferred Reporting Items for Systematic Reviews and Meta‐Analyses) guidelines, as described by Page et al. [19]. The risk of bias in the preclinical animal studies included in the review was assessed using the SYRCLE tool, as proposed by Hooijmans et al. [20].

### 2.1 Search Strategy

The literature search strategy was conducted with the aim of identifying relevant preclinical studies using terms related to the intervention of interest and the pathological condition under investigation. Searches were conducted in the electronic databases PubMed, ScienceDirect, and Scopus. The search strategy combined the term referring to the intervention of interest with terms related to colitis and inflammatory bowel disease, using the Boolean operators AND and OR, according to the following strategy: (“propolis”) AND (“colitis” OR “ulcerative colitis” OR “inflammatory bowel disease” OR “IBD”). The terms were searched in the title and/or abstract fields, depending on the features available in each database. In addition to searches of the electronic databases, the reference lists of the identified studies were manually screened to locate potentially relevant articles that had not been retrieved through the electronic search strategy.

### 2.2 Study Selection Process

Studies published through June 2025 were considered. Records identified in the databases and through additional reference searches were screened based on their titles and abstracts, and those unrelated to the objective of the review were excluded. Subsequently, potentially eligible articles were assessed in full text. Articles with full‐text availability were included if they described in vivo preclinical studies using animal models of colitis and evaluating the effects of propolis or its main bioactive compounds. At this stage, studies were excluded if they did not use animal models of colitis, did not report parameters related to the therapeutic effects or possible mechanisms of action of the intervention, or did not provide information on the origin or source of the propolis used. At the end of the selection process, 34 articles were included in this review (Figure 1).

**FIGURE 1:**
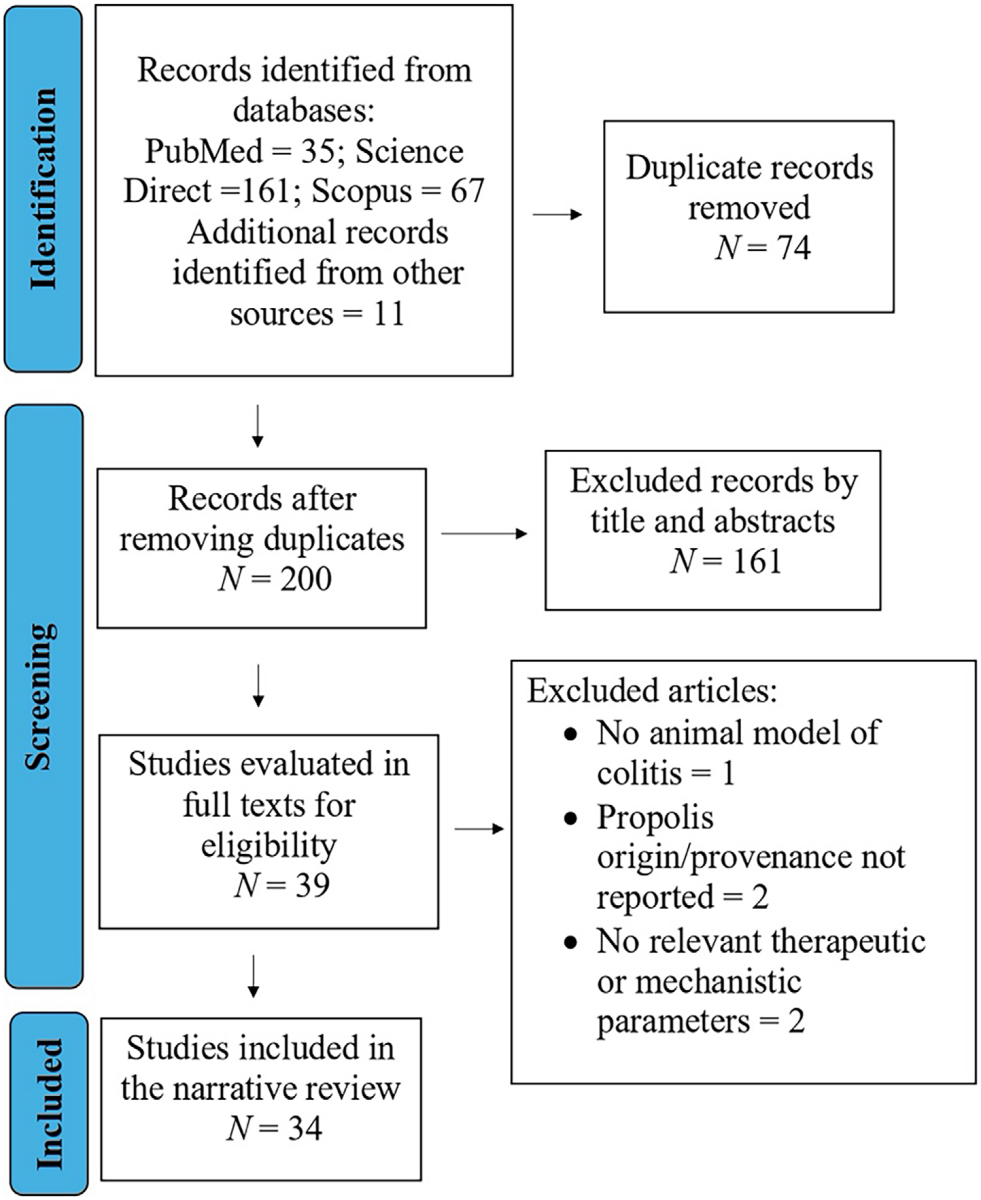
PRISMA flow diagram representing the process of identification, screening, eligibility, and inclusion of studies selected for the review. Adapted from Page et al. [19].

### 2.3 Assessment of Risk of Bias

The risk of bias in the included studies was assessed using the SYRCLE risk‐of‐bias tool for animal studies [20]. The assessment aimed to analyze the risk of bias in preclinical studies investigating the effects of propolis and its main chemical components in animal models of ulcerative colitis. The SYRCLE tool comprises 10 items distributed across different domains of bias: (1) sequence generation; (2) baseline characteristics; (3) allocation concealment; (4) random housing of animals; (5) blinding of caregivers and/or researchers; (6) random outcome assessment; (7) blinding of outcome assessors; (8) incomplete outcome data; (9) selective reporting of outcomes; and (10) other sources of bias (Figure 2 and Table S1).

**FIGURE 2:**
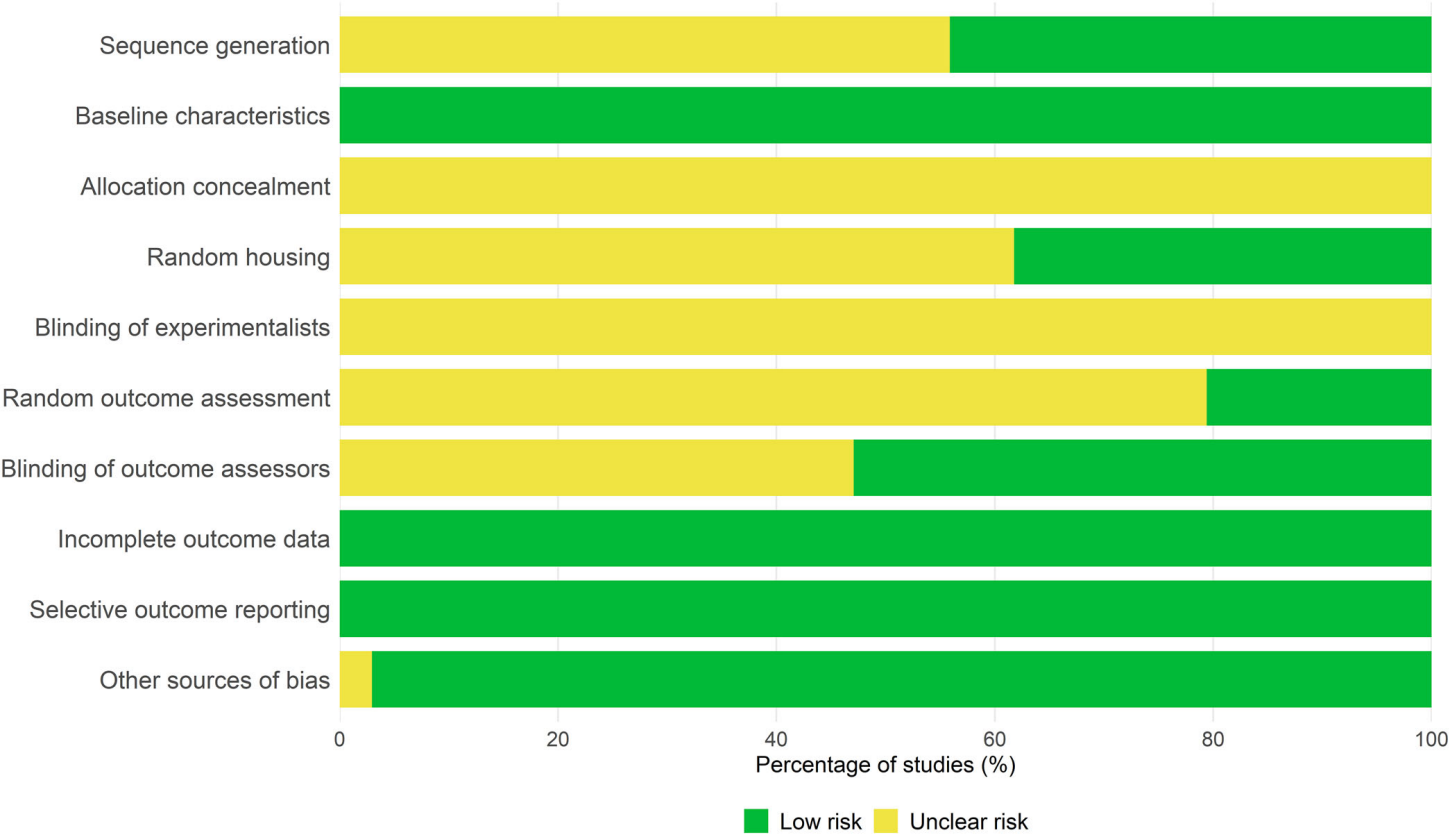
Risk of bias assessment of the included studies using the SYRCLE tool. The bars represent the percentage of the 34 studies classified as having a low or unclear risk of bias across each assessed domain.

## 3 Propolis and its Chemical Composition

Propolis, also known as “bee glue”, is a resinous substance collected by worker bees from various plant sources in their environment [21]. It is primarily used to protect the hive by sealing cracks, defending against predators and microorganisms, and providing thermal insulation [22].

The chemical composition of propolis is highly complex and varies according to geographic location and season. This intrinsic variability poses challenges for its standardization and quality control. Propolis can be classified into different types based on its botanical origin and geographical source. The most widely recognized types include poplar propolis (found in New Zealand, Europe, North America, and parts of Asia), green propolis (derived from rosemary), Brazilian propolis, birch propolis (Russia), red propolis (Cuba, Brazil, and Mexico), Mediterranean propolis (Sicily, Greece, Crete, and Malta), and Pacific propolis (Okinawa, Taiwan, and Indonesia) [21].

Propolis is composed primarily of resins (approximately 50%), wax (30%), essential oils (10%), pollen (5%), and other organic compounds (5%) [23]. Its polyphenol content is commonly used as a quality indicator [21]. The main polyphenols include flavonoids, phenolic acids, esters, phenolic aldehydes, and ketones. Capillary zone electrophoresis has identified several flavonoids in propolis extracts, including pinocembrin, acacetin, chrysin, rutin, luteolin, kaempferol, apigenin, myricetin, catechin, naringenin, galangin, and quercetin, as well as phenolic acids such as caffeic acid and cinnamic acid, and the stilbene derivative resveratrol [24]. Terpenes are responsible for the characteristic resinous aroma of propolis and can serve as markers for distinguishing between different types, as well as indicators of quality [21].

In addition, propolis contains vitamins (B1, B2, B6, C, and E) and essential minerals, including magnesium (Mg), calcium (Ca), potassium (K), sodium (Na), copper (Cu), zinc (Zn), manganese (Mn), and iron (Fe). It also contains various hydrocarbons (e.g., alkanes, alkenes, alkadienes, monoesters, diesters, and aromatic esters), and enzymes such as succinate dehydrogenase, glucose‐6‐phosphatase, adenosine triphosphatase, and acid phosphatase [25].

The extraction method significantly influences the chemical composition and biological activity of propolis. Common solvents include water, ethanol, methanol, chloroform, dichloromethane, ether, and acetone; however, ethanol—particularly at concentrations of 70%–75%—is generally considered the most effective. The bioactivity of propolis varies depending on its geographic origin, seasonal variation, and time of collection [26]. Due to its widespread use in traditional medicine, propolis has been extensively investigated in pharmacological and chemical studies [27].

Although propolis has been used since ancient times, its application expanded significantly after World War I and World War II, alongside increased scientific interest in its chemical composition and pharmacological properties. These studies have enabled differentiation of propolis types based on their chemical profiles and biological activities associated with geographic origin. Numerous investigations have demonstrated its diverse pharmacological properties, including antioxidant, anti‐inflammatory, antimicrobial, anticancer, analgesic, antidepressant, anxiolytic, and immunomodulatory effects [27, 28, 29].

## 4 Propolis and its Effect on Ulcerative Colitis

Evidence from preclinical studies indicates that propolis is a promising candidate for the treatment of UC due to its antioxidant properties and its ability to modulate the immune response and gut microbiome [30]. Experimentally induced UC models are widely used in research, employing agents such as dextran sodium sulfate (DSS), acetic acid, oxazolone, and 2,4,6‐trinitrobenzenesulfonic acid (TNBS) combined with ethanol. These models are well established and validated for reproducing key pathophysiological features of the disease [31].

Administration of propolis in animal models has been shown to reduce both macroscopic and histopathological scores of colonic damage, as well as iNOS expression, while modulating inflammatory responses [32]. Table 1 summarizes preclinical studies investigating the effects of propolis from different botanical and geographical origins in the treatment of UC (see also Figure 3).

**TABLE 1:** Summary of preclinical studies evaluating the effects of propolis from different botanical origins in ulcerative colitis.

Origin	Type of extract	Dose	Route	Duration	Sample sizes	Method	Bioactivity	Mechanism	Refs.
Egyptian propolis	Ethanolic extract	600 mg/kg: Coadministration of thymoquinone and propolis in liposomal formulations	Oral	10 days	Male Sprague‐Dawley rats (n = 60)	4% acetic acid‐induced colitis	Anti‐inflammatory and antioxidant	Reduction in MDA, TNF‐α, and nitrite levels, along with downregulation of caspase‐3 expression.	[37]
	Methanolic extract	50 and 100 mg/kg	Oral	11 days	Male and female Sprague Dawley rats (n = 40)	4% acetic acid‐induced colitis	Anti‐inflammatory and antioxidant	Reduction in MDA levels, MPO activity, NO levels, and TNF‐α levels, along with inhibition of COX‐1 and COX‐2 enzymes.	[38]
Chinese propolis	Ethanol extract	0.3% diet	Oral	21 days	Male Sprague Dawley rats (n = 40)	3% DSS‐induced colitis	Modulation of the intestinal microbiota	Increased intestinal microbial diversity, including enrichment of bacteria from the phyla Proteobacteria and Acidobacteria.	[51]
	Ethanol extract	300 mg/kg	Oral	17 days	Male Sprague Dawley rats (n = 32)	3% DSS‐induced colitis.	Anti‐inflammatory; antioxidant and modulation of intestinal microbiota	Reduction in MDA levels and increased T‐AOC, along with suppression of colonic inflammatory markers (IL‐1β, IL‐6, and MCP‐1) and enhanced gut microbiota diversity.	[33]
	Water‐soluble extract	50, 100 and 200 mg/kg	Oral	7 days	Male Sprague Dawley rats (n = 48)	4% DSS‐induced colitis	Anti‐inflammatory	Reduction in MDA, IL‐6, and TNF‐α levels, along with decreased TRPV1 expression.	[34]
	Water‐soluble extract	50, 100 and 200 mg/kg	Oral	7 days	Male Sprague Dawley rats (n = 48)	4% DSS‐induced colitis	Anti‐inflammatory	Reduction in the expression of PKC, TRPV1, CGRP and SP.	[21]
	Water‐soluble extract	50, 100 and 200 mg/kg	Oral	7 days	Male Sprague Dawley rats (n = 48)	5% DSS‐induced colitis.	Anti‐inflammatory and antioxidant	Increased activity of SOD and GSH‐Px, reduced ROS levels, and decreased pro‐inflammatory cytokines (TNF‐ɑ, IL‐6, and IL‐9).	[35]
Korean Propolis	Ethanol extract	Extract diluted in drinking water in proportions of 1:1000 and 1:500	Oral	17 days	Female BALB/c mice (undefined sample)	2% DSS‐induced colitis	Anti‐inflammatory	Reduction in the inflammatory cytokine KC, decreased infiltration of immune cells T cells (CD3), B cells (CD20), and neutrophils (MPO), and reduced expression of inflammatory markers such as COX‐2. Additionally, attenuation of goblet cell loss and decrease antibody reactivity to inflammatory markers.	[39]
Brazilian Propolis	Ethanol extract (green propolis)	300 mg/kg	Oral	17 days	Male Sprague Dawley rats (n = 32)	3% DSS‐induced colitis.	Anti‐inflammatory; antioxidant and modulation of intestinal microbiota	Reduction in MDA and CAT levels, suppression of colonic inflammatory markers (IL‐1β, IL‐6, and MCP‐1), increased TGF‐β expression, and reduced abundance of *Bacteroides* spp.	[33]
	Ethanolic extract (green propolis)	0.3% diet	Oral	9 days	Male BALB/c mice (n = 21)	2% DSS‐induced colitis	Anti‐inflammatory	Reduction in IL‐17a expression.	[42]
	Ethanolic extract (green propolis)	6, 7 and 20 mg/g diet	Oral	18 days	Male BALB/c mice (n = 18)	TNBS‐induced colitis	Anti‐inflammatory	Reduction in the frequency of IFNγ‐producing CD4^+^ T cells under Th1‐polarizing conditions, along with inhibition of Th1 differentiation.	[41]
	Ethanolic extract (green propolis)	300 mg/kg	Oral	12 days	Male C57BL/6 mice (n = 30)	2% DSS‐induced colitis	Anti‐inflammatory	Reduction in TNF‐α, IL‐6, IL‐1β levels and ROS production.	[40]
	Hydroalcoholic extract (green propolis)	300 mg/kg	Oral	7 days	Female Swiss mice (n = 40)	3% DSS‐induced colitis	Antioxidant	Increased mucin levels, along with normalization of SOD activity and GSH levels.	[46]
	Hydroalcoholic extract	8% (w/w)	Rectal	12 days	Male Wistar albino rats (n = 50)	TNBS‐induced colitis	Antioxidant	Increase in MPO	[48]
	Hydroalcoholic extract (brown propolis)	100 and 300 mg/kg	Oral	5 days	Female Wistar rats (n = 30)	TNBS‐induced colitis	Antioxidant	Reduction in MDA levels, restoration of GSH levels, and increased activity of SOD, CAT and GST in the colon, along with inhibition of MPO activity and reduced neutrophil chemotaxis and migration.	[47]
	Hydroalcoholic extract (red propolis)	10 and 100 mg/kg	Oral	7 days	Male Wistar rats (n = 20)	4% acetic acid‐induced colitis	Antioxidant	Reduction in MPO activity and decreased iNOS expression.	[43]

Abbreviations: CAT (catalase); COX‐1 (cyclooxygenase‐1); COX‐2 (cyclooxygenase‐2); DSS (dextran sulfate sodium); GSH (glutathione); GSH‐Px (glutathione peroxidase); GST (glutathione‐S‐transferase); IFNγ (gamma interferon); IL (cytokines); iNOS (inducible nitric oxide synthase); MCP‐1 (monocyte chemoattractant protein‐1); MDA (malondialdehyde); MPO (myeloperoxidase); NO (nitric oxide); ROS (reactive oxygen species); SOD (superoxide dismutase); T‐AOC (total antioxidant capacity); TGF‐β (transforming growth factor‐β);TH1 (type 1 T helper cell); TNBS (Trinitrobenzenesulfonic acid); TNF‐α (tumor necrosis factor‐alpha); TRPV1 (transient receptor potential vanilloid 1).

**FIGURE 3:**
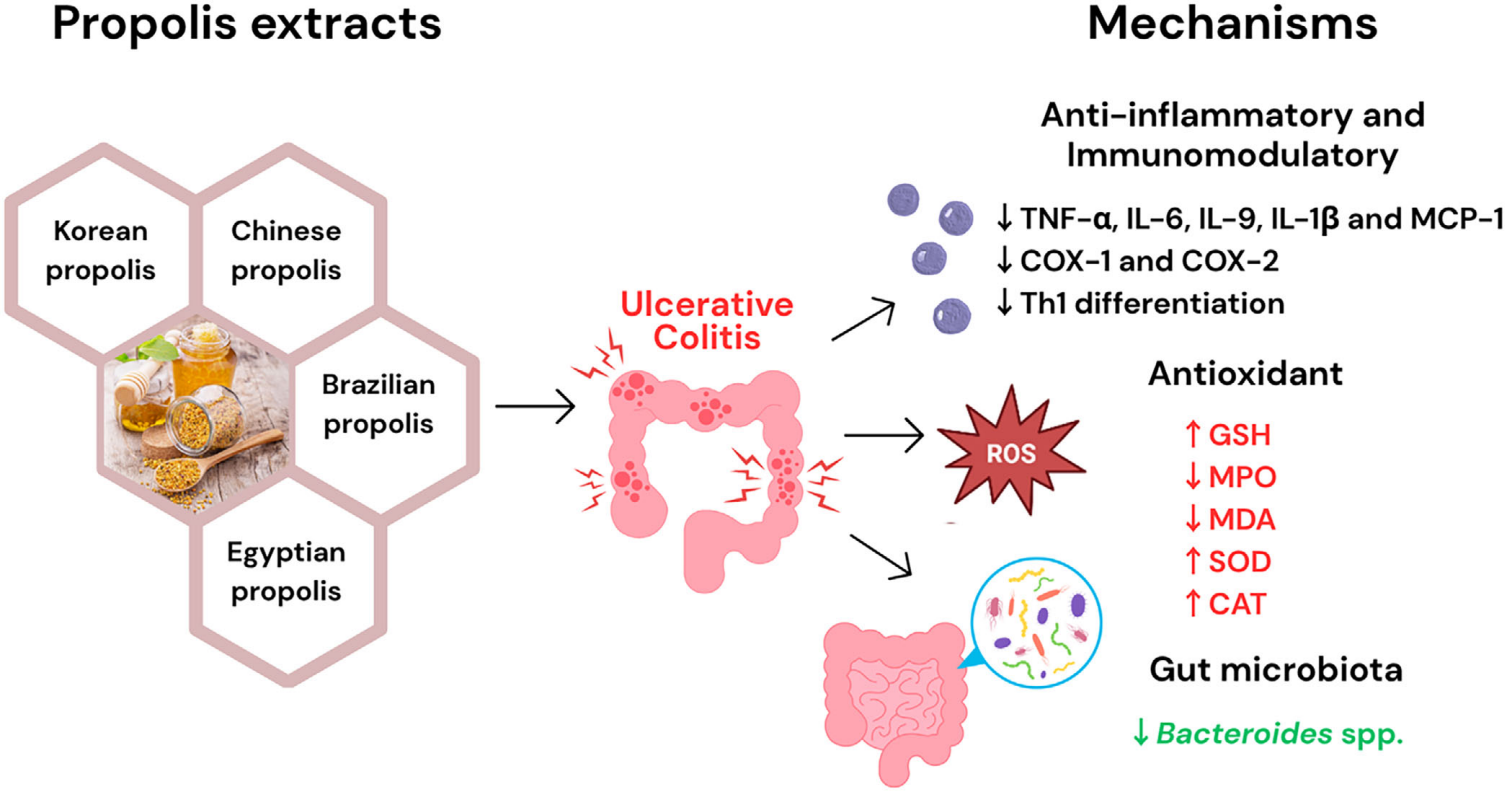
Botanical sources of propolis and their relevance in the treatment of ulcerative colitis. Created by the authors using Canva.

### 4.1 Anti‐Inflammatory and Immunomodulatory Activity

UC is characterized by persistent inflammation of the colonic mucosa, associated with overactivation of the transcription factor NF‐κB, which leads to increased production of pro‐inflammatory cytokines such as IL‐1β, IL‐6, TNF‐α, and interferons [32]. In this context, propolis—owing to its anti‐inflammatory and immunomodulatory properties—may play an important role in regulating immune system components [18].

In vivo studies using DSS‐induced colitis in rats have demonstrated that Chinese propolis significantly reduces levels of pro‐inflammatory cytokines, including TNF‐α, IL‐6, IL‐9, IL‐1β, and MCP‐1. Additionally, a reduction in the TRPV1 receptor in colonic tissue has been observed, contributing to the attenuation of intestinal inflammation associated with UC [33, 34, 35]. TRPV1 is considered a key mediator of visceral pain and inflammation, and its inhibition has been associated with the alleviation of T cell‐mediated colitis symptoms [36]. In this context, Qian et al. [31] investigated the involvement of the protein kinase C‐transient receptor potential cation channel subfamily V member 1‐calcitonin gene‐related peptide/substance P (PKC‐TRPV1‐CGRP/SP) signaling pathway. Their findings suggest that the therapeutic effects of Chinese propolis in UC may be related to inhibition of this pathway, resulting in reduced release of inflammatory mediators and subsequent alleviation of intestinal inflammation.

Combined therapy using liposomal formulations of thymoquinone and Egyptian propolis in animals with acetic acid‐induced colitis has shown significant therapeutic effects, including reduced levels of TNF‐α and nitrite, as well as downregulation of caspase‐3 expression [37]. Egyptian propolis has also demonstrated a potent inhibitory effect on the inflammatory enzymes cyclooxygenase‐1 (COX‐1) and cyclooxygenase‐2 (COX‐2). Notably, selective inhibition of COX‐2—an enzyme that plays a crucial role in prostaglandin biosynthesis and in maintaining colonic mucosal integrity under inflammatory conditions—has been reported [38].

Hwang et al. [39] reported that Korean propolis significantly reduces the severity of colitis in mice with DSS‐induced colitis. This effect was associated with decreased levels of the inflammatory cytokine KC (a functional homolog of human IL‐8), reduced infiltration of immune cells—including T cells (CD3), B cells (CD20), and neutrophils—and downregulation of COX‐2. Additionally, Korean propolis attenuated goblet cell loss and decreased antibody reactivity to inflammatory markers in colonic tissue [39].

Brazilian green propolis is one of the most extensively studied types in recent years [21]. Experimental studies have demonstrated its potential to modulate immune responses, cytokine production, and inflammation. Treatment with ethanolic extract of Brazilian green propolis (300 mg/kg) significantly reduced levels of inflammatory cytokines such as TNF‐α, IL‐6, and IL‐1β [40]. Furthermore, it suppressed the expression of the inflammatory marker MCP‐1 and increased the expression of TGF‐β in the colon of mice with DSS‐induced acute colitis [33].

T helper 1 (Th1) cells play a key role in several autoimmune and inflammatory diseases, including IBD. Okamoto et al. [41] demonstrated that ethanolic propolis extract reduces the frequency of IFN‐γ‐producing CD4^+^ T cells under Th1‐polarizing conditions, thereby suppressing Th1 differentiation and contributing to the improvement of TNBS‐induced colitis in mice. Moreover, IL‐17 expression is upregulated in the blood and tissues of patients with IBD. Shimizu and Suzuki [42] showed that ethanolic propolis extract suppresses IL‐17 differentiation in mice with DSS‐induced colitis, which may represent an additional mechanism underlying the attenuation of disease severity. The presence of cinnamic acid derivatives, such as artepillin C, likely contributes—at least in part—to these protective effects of propolis.

### 4.2 Antioxidant Activity

The maintenance of intestinal mucosal integrity relies, in part, on endogenous antioxidant defense systems that counteract the production of reactive oxygen species (ROS), including superoxide dismutase (SOD), catalase (CAT), and glutathione peroxidase (GPx). Several studies have shown that UC is associated with excessive ROS production, leading to lipid peroxidation and amplification of inflammatory responses [43]. Both in vitro and in vivo evidence supports the potential of propolis as a natural agent capable of mitigating oxidative stress [44].

Oxidation of unsaturated lipids leads to the formation of by‐products, such as malondialdehyde (MDA), a well‐established biomarker of oxidative stress in UC [45]. Studies using ethanolic and methanolic extracts of Egyptian propolis have demonstrated reduced MDA levels in animals with DSS‐induced colitis [37, 38]. The methanolic extract also reduced the activity of myeloperoxidase (MPO), an enzyme produced by neutrophils that catalyzes the formation of highly reactive hypochlorous acid from chloride ions [38, 43].

The protective effects of Chinese propolis have also been investigated in experimental models of UC. Treatment with water‐soluble extract resulted in reduced ROS levels, accompanied by increased activity of endogenous antioxidant enzymes such as SOD and GPx, which contribute to suppression of neutrophil activity [35]. Similarly, Wang et al. [33] reported that ethanolic propolis extract enhanced resistance to DSS‐induced oxidative stress in the colon, as evidenced by decreased MDA levels and increased total antioxidant capacity (T‐AOC).

Ethanolic extracts of Brazilian green propolis have also demonstrated antioxidant effects, including reduced MDA levels and modulation of CAT activity [33]. In addition, hydroalcoholic extracts promoted normalization of SOD activity and glutathione (GSH) levels, although no significant effect on MPO activity in the colon of DSS‐induced colitis models was observed [46].

The hydroalcoholic extract of Brazilian brown propolis significantly reduced MDA levels and restored GSH levels, as well as the activity of antioxidant enzymes such as SOD, CAT, and glutathione S‐transferase (GST) in the colon of rats with TNBS‐induced colitis [47].

Finally, hydroalcoholic extract of Brazilian red propolis was also evaluated in an acetic acid‐induced colitis model in rats. Treatment was associated with reduced MPO activity, improved macroscopic and histopathological scores of tissue damage, and decreased expression of iNOS [43]. In contrast, Gonçalves et al. [48] reported an increase in MPO activity following treatment with a hydroalcoholic propolis extract in a rat model of TNBS‐induced colitis. This discrepancy may be related to differences between the experimental models and the characteristics of the therapeutic approaches employed. Gonçalves et al. [48] used a TNBS‐induced colitis model in rats, whereas Barbosa Bezerra et al. [43] used acetic acid to induce colitis. Furthermore, in Gonçalves et al. [46], treatment was initiated 48 h after colitis induction, when the inflammatory process had already been established, thus constituting a therapeutic intervention. In contrast, Barbosa Bezerra et al. [43] adopted a preventive approach, administering propolis before colitis induction. This difference in the timing of intervention and in the experimental models may have influenced the inflammatory response and, consequently, MPO activity.

### 4.3 Modulatory Effects on Gut Microbiota

Accumulating evidence indicates that reduced diversity of the fecal microbiota is one of the most consistent features of UC [49]. Alterations in gut microbiota composition can lead to an overgrowth of pathogenic bacteria, which release enterotoxins that increase intestinal permeability and produce immunomodulatory factors that disrupt immune homeostasis. This imbalance facilitates bacterial invasion and direct damage to intestinal epithelial cells, compromising the integrity of the mucosal barrier. As barrier function deteriorates, bacterial translocation increases, further exacerbating mucosal damage and perpetuating a cycle of inflammation and tissue injury [50].

Treatment with Chinese propolis has been shown to alleviate DSS‐induced colitis in rats fed a Western diet, promoting a significant increase in gut microbiota diversity, including enrichment of bacteria from the phyla Proteobacteria and Acidobacteria [51]. Additionally, both Chinese and Brazilian propolis have been reported to significantly reduce populations of *Bacteroides* spp., highlighting their modulatory effect on the gut microbiota [33].

In other studies, Chinese propolis has demonstrated the ability to modulate the gut microbiota composition and improve intestinal barrier function in diabetic rat models [52]. Similarly, Brazilian green propolis has been shown to modulate the gut microbiota, reduce endotoxemia, and suppress activation of the TLR4 signaling pathway in mice fed a high‐fat diet [53].

## 5 Bioactive Compounds of Propolis in the Treatment of UC

Propolis is rich in polyphenolic constituents, with flavonoids recognized as its main bioactive components. These compounds have been investigated in preclinical studies for the treatment of UC (Table 2 and Figure 4).

**TABLE 2:** Main bioactive compounds of propolis in their therapeutic effects in ulcerative colitis.

Compound	Dese	Route	Duration	Sample sizes	Method	Bioactivity	Mechanisms	Refs.
Caffeic acid phenethyl ester (CAPE)	30 mg/kg body weight	Intraperitoneal injection	7 days	Female Lewis rats (undefined sample)	Peptidoglycan‐polysaccharide (PG‐PS)‐induced colitis	Anti‐inflammatory	Reduction in NF‐kB activation and decreased levels of TNF‐α and IL‐1β.	[54]
	30 mg/kg body weight	Intraperitoneal injection	7 days	Female C57BL/6 mice (undefined sample)	2.5% DSS‐induced colitis	Anti‐inflammatory	Reduction in MPO activity, INF‐γ, IL 6, IL 1‐β, TNF‐α, and IL 10, along with decreased p65 expression in the NF‐κB pathway.	[55]
	10 and 45 mg/kg body weight (Emulsion)	Oral	22 days	Male C57BL6/J mice (n = 50)	3% DSS‐induced colitis	Anti‐inflammatory and modulation of intestinal microbiota	Reduction in IL‐1β, IL‐6, IL‐10, and TGF‐β levels, as well as P65 expression in the NF‐κB signaling pathway; attenuation of short‐chain fatty acid loss and modulation of gut microbiota imbalance.	[56]
3'‐hydroxypterostilbene (trans‐3,5‐dimethoxy‐3',4'‐dihydroxystilbene)	50 and 250 mg/kg diet	Oral	10 weeks	Male ICR mice (undefined sample)	Azoxymethane (AOM)/ 2% DSS‐induced colitis‐associated cancer	Anti‐inflammatory	Reduction in iNOS, COX‐2, and IL‐6 levels, along with inhibition of the IL‐6/STAT3 signaling pathway and restoration of suppressor of cytokine signaling 3 levels in colonic tissue.	[57]
Pinobanksin	10, 20 and 40 mg/kg	Oral	9 days	C57BL/6 mice (undefined sample)	2.5% DSS‐induced colitis	Antioxidant	Inhibition of ferroptosis and increased GSH biosynthesis.	[60]
Pinocembrin	5 and 10 mg/kg body weight	Oral	14 days	Male Sprague‐Dawley rats (n = 30)	3.5% DSS‐induced colitis	Anti‐inflammatory, modulation of microbiota, and restoring intestinal barrier	Increased expression of TGF‐β, ZO‐1, and occludin, along with reduced mRNA levels of IL‐1β, IL‐6 and TNF‐α; decreased loss of short‐chain fatty acids and increased gut microbiota diversity.	[61]
Galangin	40 mg/kg body weight	Oral	4 weeks	Male Swiss albino mice (undefined sample)	3% DSS‐induced colitis	Anti‐inflammatory and antioxidant	Reduction in IL‐6 and TNF‐α levels, decreased TLR4 receptor expression, and suppression of NF‐κB p65 activation; increased GSH levels and reduced MDA and LDH levels.	[62]
Quercetin	1 mg/kg/day	Oral	15 days	Female Wistar rats and male BALB/c mice (n = 32)	2%–5% DSS‐induced colitis	Anti‐inflammatory	Reduction in MPO activity and inhibition of TNF‐α, IL‐1β and iNOS expression, associated with suppression of the NF‐κB pathway.	[63]
	1, 10 and 100 mg/kg (Quercetin‐loaded microcapsules)	Oral	N/D	Male Swiss mice (undefined sample)	7.5% acetic acid‐induced colitis	Anti‐inflammatory and antioxidant	Reduction in IL‐1β and IL‐33 production; prevention of IL‐10 and GSH depletion; increased antioxidant capacity (ABTS and FRAP assays).	[64]
	50 and 100 mg/kg	Oral	11 days	Male Wistar rats (n = 36)	TNBS‐induced colitis	Antioxidant	Reduction in MPO activity, MDA, and NO levels, along with increased GSH levels.	[65]
	50 and 100 mg/kg	Oral	5 days	Male Wistar rats (n = 30)	3% acetic acid‐induced colitis	Antioxidant	Reduction in MPO activity, MDA, and NO levels, along with increased GSH levels.	[66]
	9 mg/kg/day (Biocompatible quercetin nanovesicles)	Oral	3 days	Male Wistar rats (undefined sample)	TNBS‐induced colitis	Antioxidant	Reduction in MPO	[67]
Kaempferol	0.30% (w/w)	Oral	21 days	Female C57BL/6J mice (n = 60)	2% DSS‐induced colitis	Anti‐inflammatory	Decreased NO and PGE_2_ levels; suppression of TNF‐α, IL‐1β and IL‐6 mRNA expression; reduction in plasma levels of LTB_4_ and MPO; and upregulation of TFF3 mRNA expression.	[68]
Luteolin	20 and 50 mg/kg	Oral	11 days	Female C57BL/6CrSlc mice (n = 20)	2% DSS‐induced colitis	Anti‐inflammatory	Inhibition of macrophage and CD4^+^ T cell infiltration into the colonic mucosa, along with reduced IFN‐γ mRNA expression.	[70]
	20 and 50 mg/kg	Oral	18 days	Male C57BL/6 mice (n = 30)	3% DSS‐induced colitis	Anti‐inflammatory and antioxidant	Decreased expression of iNOS, TNF‐α, and IL‐6; reduced MDA levels; increased activity of SOD and CAT; and upregulation of *Nrf2* and its downstream targets, HO‐1 and NQO1.	[69]
Chrysin	25 mg/kg of body weight	Oral	7 days	Female C57BL mice (undefined sample)	4% DSS/ TNBS‐induced colitis	Anti‐inflammatory	Modulation of the PXR/NF‐κB signaling pathway; inhibition of iNOS, ICAM‐1, MCP‐1, Cox2, TNF‐α, and IL‐6; and reduction in MPO activity.	[72]
Naringenin	0.3% (w/w)	Oral	9 days	Male BALB/c mice (n = 82)	2% DSS‐induced colitis	Anti‐inflammatory	Reduction in IFNγ, IL‐6, MIP‐2 and IL‐17A levels, with suppression of IL‐6 and IL‐17A expression; increase expression of occludin, JAM‐A and claudin‐3.	[75]
	50 mg/kg	Oral	10 days	Female C57BL mice (undefined sample)	4% DSS‐induced colitis	Anti‐inflammatory	Inhibition of TLR4 receptor mRNA and protein expression, blockade of the NF‐κB signaling pathway, and suppression of iNOS, ICAM‐1, MCP‐1, Cox2, TNF‐α, and IL‐6.	[73]
	25, 50 and 100 mg/kg/day	Oral	7 days	Male Wistar albino rats (n = 42)	4% acetic acid‐induced colitis	Anti‐inflammatory and antioxidant	Reduction in TBARS levels; increased T‐GSH and NP‐SH levels, as well as and CAT and SOD activity; increased DNA, RNA and total protein levels; and inhibition of TNF‐α, IL‐1β and IL‐6, PGE_2,_ and NO production.	[74]

Abbreviations: ABTS (2,2'‐azinobis (3‐ethylbenzothiazoline‐6‐sulfonic acid)); AOM (azoxymethane); CAT (catalase); COX‐2 (cyclooxygenase‐2); DNA and RNA (nucleic acids); DSS (dextran sulfate sodium); FRAP (Ferric Reducing Ability); GSH (glutathione peroxidase); GSH (glutathione); ICAM‐1 (Intercellular Adhesion Molecule 1); IFNγ (gamma interferon); IL (cytokines); iNOS (inducible nitric oxide synthase); JAM‐A (Junctional Adhesion Molecule‐A); LTB4 (leukotriene B4); MCP‐1 (monocyte chemoattractant protein‐1); MDA (malondialdehyde); MPO (myeloperoxidase); mRNA (messenger RNA); NF‐κB (nuclear factor kappa B); NO (nitric oxide); NP‐SH (nonprotein sulfadryls); NQO1 (quinone oxidoreductase 1); Nrf2 (Nuclear factor erythroid 2‐related factor 2); PGE2 (prostaglandin); ROS (reactive oxygen species); SOD (superoxide dismutase); TBARS (thiobarbituric acid‐reactive substances); TFF3 (Trefoil Factor Family); TGF‐β (transforming growth factor‐β); T‐GSH (total glutathione sulfadryls); TLR4 (Toll‐like receptor 4); TNBS (Trinitrobenzenesulfonic acid); TNF‐α (tumor necrosis factor‐alpha).

**FIGURE 4:**
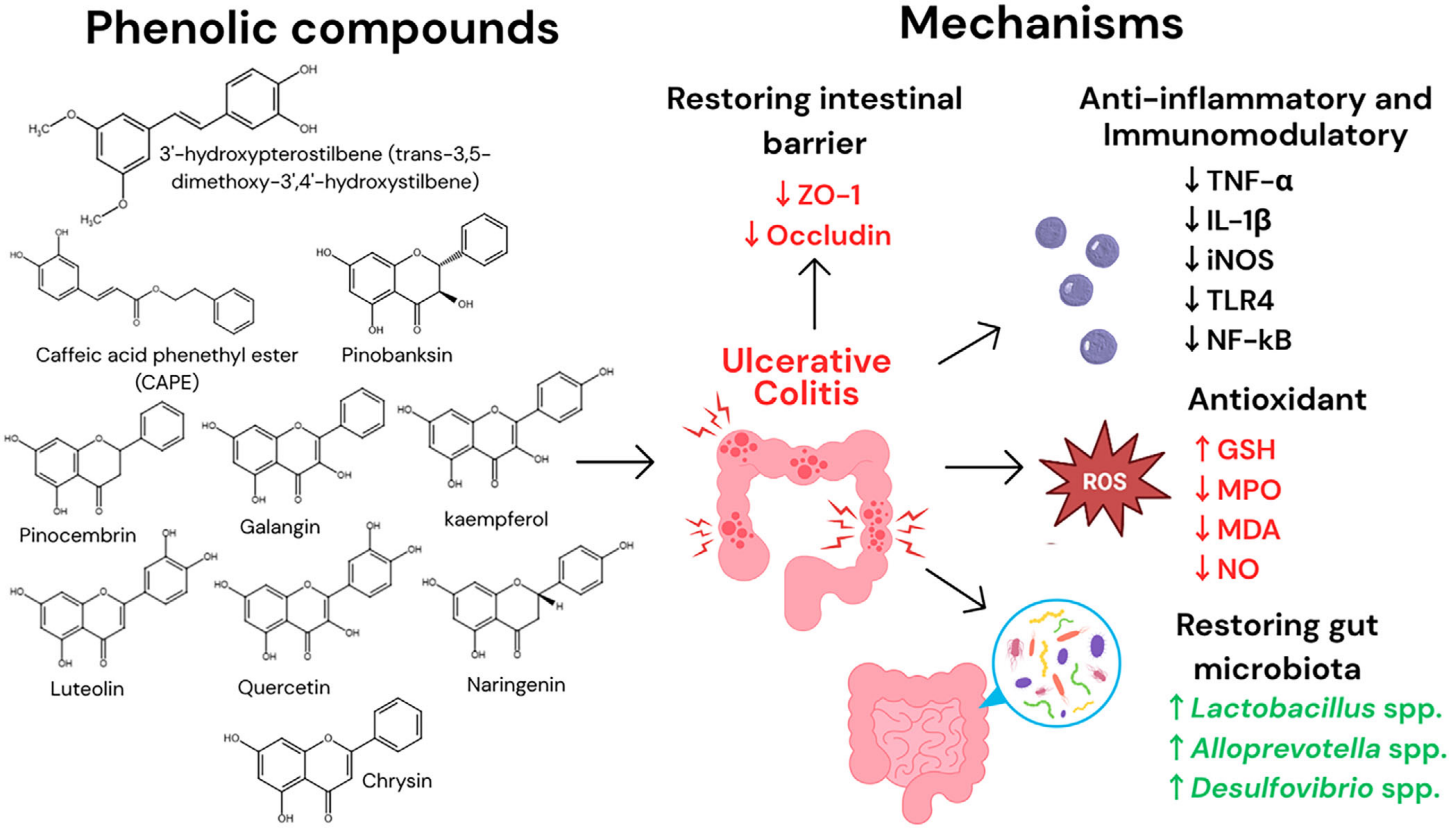
Chemical structures and biological activities of propolis‐derived compounds in ulcerative colitis. Created by the authors using Canva.

Caffeic acid phenethyl ester (CAPE) is a phenolic compound derived from propolis with well‐documented anti‐inflammatory activity. Its effects have been evaluated in a peptidoglycan‐polysaccharide (PG‐PS)‐induced colitis model in rats. CAPE attenuated colonic inflammation by inhibiting the NF‐κB signaling pathway and promoting apoptosis in macrophages [54]. In addition, this compound has been shown to reduce MPO activity and decrease levels of pro‐inflammatory cytokines, including INF‐γ, IL6, IL1‐β, and TNF‐α [55].

The therapeutic potential of CAPE has also been explored using a solid‐in‐oil‐in‐water (S/O/W) emulsion delivery system. This formulation effectively downregulated excessive inflammatory cytokine production in colonic tissue and inhibited p65 expression within the NF‐κB pathway. Furthermore, it enhanced the production of short‐chain fatty acids and regulated gut microbiota composition by increasing microbial diversity, restoring beneficial bacteria such as *Odoribacter*, and reducing potentially harmful genera such as *Afipia* and *Sphingomonas* [56].

The compound 3'‐hydroxypterostilbene (trans‐3,5‐dimethoxy‐3',4'‐hydroxystilbene), found in propolis and in plant species such as *Sphaerophysa salsula* and *Pterocarpus marsupium*, has been investigated for its chemopreventive effects in colitis‐associated cancer using the AOM/DSS model. Administration of this compound resulted in reduced expression of iNOS, COX‐2, and IL‐6, as well as significant inhibition of the IL‐6/STAT3 signaling pathway. Additionally, it restored suppressor of cytokine signaling 3 (SOCS3) protein levels in colonic tissue [57].

Ferroptosis, a form of iron‐induced cell death characterized by lipid peroxidation and iron accumulation, has been implicated in the pathophysiology of IBD [58, 59]. Pinobanksin, a flavonoid derived from propolis, has demonstrated significant inhibitory effects on ferroptosis in colon epithelial cells isolated from mice with DSS‐induced colitis. These effects are primarily mediated through activation of glutathione peroxidase 4 (GPX4), a key negative regulator of ferroptosis. Additionally, activation of cystine transporter SLC7A11 by pinobanksin enhances cystine uptake and promotes GSH biosynthesis [60].

Pinocembrin, another flavonoid commonly found in propolis, exhibits potent protective effects on the gastrointestinal tract. Experimental studies have shown that it alleviates DSS‐induced colitis in rats. Pre‐treatment with pinocembrin (5 and 10 mg/kg) modulated inflammatory responses by increasing colonic expression of TGF‐β and reducing mRNA levels of pro‐inflammatory cytokines such as IL‐1β, IL‐6, and TNF‐α. Additionally, it enhanced the expression of tight junction proteins, including ZO‐1 and occludin, thereby promoting epithelial barrier integrity and mucosal repair. Pinocembrin also restored levels of short‐chain fatty acids and improved gut microbial diversity, with recovery of beneficial genera such as *Lactobacillus*, *Alloprevotella*, and *Desulfovibrio* [61].

The therapeutic effect of the flavonoid galangin, a promising bioactive compound present in bee propolis, has been investigated in a DSS‐induced colitis model in mice. Treatment with galangin significantly improved the histopathological alteration and reduced tissue damage. These effects were associated with decreased expression of Toll‐like receptor 4 (TLR4) and suppression of NF‐κB p65 activation. In addition, reductions in the levels of inflammatory cytokines such as IL‐6 and TNF‐α were observed, along with antioxidant effects, evidenced by increased GSH levels and decreased levels of MDA and lactate dehydrogenase (LDH) [62].

The flavonoid quercetin has also demonstrated potential for inhibiting the NF‐κB pathway. In the study by Comalada et al. [63], the in vivo effects of quercitrin (the glycosylated form of quercetin) in a DSS‐induced colitis model in rats were shown to depend on its conversion to quercetin following glycoside cleavage by the gut microbiota. This is supported by evidence that quercetin, but not quercitrin, inhibits the expression of TNF‐α, IL‐1β, and iNOS by suppressing NF‐κB activation without affecting c‐Jun N‐terminal kinase activity (in vitro and in vivo). Furthermore, quercetin has been widely studied in different experimental models of colitis, including acetic acid‐induced colitis in mice [64, 65] and TNBS‐induced colitis in rats [66, 67]. In these models, quercetin administration attenuated colonic damage, as evidenced by reduced MPO activity, MDA and NO levels, and increased GSH levels, indicating both antioxidant and anti‐inflammatory effects.

Other flavonoids found in propolis, such as kaempferol, luteolin, chrysin, and naringenin, have also been investigated for their therapeutic potential in UC. Kaempferol treatment has been shown to mitigate DSS‐induced mucosal damage in mice, an effect attributed to its anti‐inflammatory properties. This included reduction in plasma levels of NO and prostaglandin E_2_ (PGE_2_), suppression of the mRNA expression of pro‐inflammatory cytokines (TNF‐α, IL‐1β, and IL‐6), decreased leukotriene B_4_ (LTB_4_) levels and MPO activity, and upregulation of TFF3 mRNA expression, a marker associated with goblet cell function [68].

Luteolin, a natural flavonoid with strong antioxidant capacity, has demonstrated protective effects in DSS‐induced colitis models. Administration of luteolin (20 and 50 mg/kg) significantly reduced the expression of inflammatory mediators such as iNOS, TNF‐α, and IL‐6, as well as colonic MDA levels. It also increased the activity of antioxidant enzymes (SOD and CAT) and upregulated the transcription factor Nrf2 and its downstream targets, hemeoxygenase‐1 (HO‐1) and NAD(P)H quinone dehydrogenase (NQO1) [69]. Additionally, luteolin reduced infiltration of macrophage and interferon‐γ‐producing CD4^+^ T cell in the colonic mucosa and decreased IFN‐γ mRNA expression [70].

In a related in vitro study, luteolin was shown to cross the Caco‐2 monolayer and act directly on intestinal immune cells. The aglycone form released at the epithelial interface inhibited NF‐κB nuclear translocation in RAW264.7 macrophages, resulting in reduced TNF‐α mRNA expression and subsequent downregulation of IL‐8 expression in Caco‐2 cells [70]. These findings highlight the importance of luteolin in its aglycone form in modulating intestinal inflammatory and immune responses in IBD.

Chrysin has been identified as a ligand of the human pregnane X receptor (PXR) in vitro [71]. PXR is a key regulator of CYP3A4 (the human homolog of rodent Cyp3a11) and is predominantly expressed in the adult hepatobiliary system. Pre‐treatment with chrysin led to downregulation of NF‐κB target genes, including iNOS, intercellular adhesion molecule‐1 (ICAM‐1), MCP‐1, COX‐2, TNF‐α, and IL‐6, in the colonic mucosa of mice with DSS‐and TNBS‐induced colitis. Moreover, chrysin activated both human and murine PXR in a dose‐dependent manner and increased the expression of PXR target genes such as Cyp3a11 and Mdr1 in the colon, but not in the liver, suggesting a gut‐specific mechanism. The findings indicate that chrysin may exert its protective effect through PXR‐mediated inhibition of NF‐κB signaling [72].

Naringenin has also demonstrated significant protective effects in experimental colitis models. Pre‐treatment with naringenin attenuated DSS‐induced colitis severity and was associated with downregulation of pro‐inflammatory mediators, including iNOS, ICAM‐1, MCP‐1, COX‐2, TNF‐α, and IL‐6 in the colonic mucosa. The reduction in TNF‐α and IL‐6 levels correlated with decreased mRNA and protein expression of the TLR4, suggesting modulation of innate immune signaling pathway [73].

In an acetic acid‐induced colitis model, naringenin also exhibited antioxidant effects, as evidenced by decreased levels of thiobarbituric acid reactive substances (TBARS), increased total glutathione (T‐GSH) and nonprotein sulfhydryl (NP‐SH) levels, and enhanced CAT and SOD activity. Additionally, increases in nucleic acid (DNA and RNA) and total protein content were observed, along with inhibition of TNF‐α, IL‐1β, IL‐6, PGE_2_, and NO production [74]. Finally, evidence suggests that naringenin may attenuate DSS‐induced colitis, at least in part, by preserving intestinal epithelial barrier integrity [75].

In summary, while these major propolis‐derived compounds exert protective effects in colitis, they exhibit both overlapping and highly distinct therapeutic mechanisms. Suppression of the NF‐κB pathway represents a shared core anti‐inflammatory axis for CAPE, galangin, quercetin, luteolin, and naringenin. However, specific targets differ; for instance, galangin and naringenin act upstream by downregulating TLR4 expression, whereas pinocembrin and naringenin uniquely reinforce the physical epithelial barrier by upregulating tight junction proteins like ZO‐1 and occludin. Furthermore, while luteolin directly drives the Nrf2/HO‐1 antioxidant cascade, pinobanksin exhibits a unique protective role by targeting GPX4‐mediated enterocyte ferroptosis. Understanding these compound‐specific pathways is crucial for designing future standardized synergetic therapies.

## 6 Current Status of Clinical Evidence on Propolis in UC

The clinical evidence regarding the use of propolis in UC remains extremely limited, particularly when compared with the body of evidence generated from preclinical studies. Although clinical studies evaluating propolis have been conducted in other gastrointestinal conditions, the available evidence cannot be directly extrapolated to UC because of differences in disease pathophysiology, study populations, interventions, and clinical outcomes.

The systematic review by Soleimani et al. [30], which evaluated the effects of propolis on inflammatory bowel disease, found that the available literature consisted predominantly of preclinical studies. In these studies, propolis administration demonstrated favorable effects on clinical, macroscopic, and histological parameters in experimental models of colitis. The authors emphasized, however, the need for clinical studies to determine the therapeutic applicability of propolis in humans. Importantly, the absence of sufficient clinical evidence prevents definitive conclusions regarding its efficacy and safety in patients with UC.

More recently, Lu et al. [76] reviewed the evidence regarding the gastroprotective effects of propolis in experimental models of inflammatory bowel disease and discussed strategies aimed at facilitating its future clinical translation. The authors emphasized that evidence of efficacy remains predominantly preclinical and identified the development of delivery systems and strategies to improve bioavailability as promising approaches for expanding the therapeutic potential of propolis.

Thus, although the body of preclinical evidence supports the therapeutic potential of propolis in UC, its clinical applicability remains uncertain. The limited availability of clinical studies specifically investigating propolis in patients with UC represents an important evidence gap and highlights the need for well‐designed clinical trials to establish its efficacy, safety, optimal dosage, and therapeutic applicability in this population.

## 7 Considerations on the Safety of Propolis use

The development of treatment agents that combine efficacy and safety remains a major goal in the management of IBD [63]. Preclinical studies have evaluated the toxicity and safety profiles of propolis extracts and their bioactive constituents. Overall, propolis exhibits low toxicity, with reported LD_50_ values ranging from 1.8 to 7.0 g/kg body weight in rats and mice [26].

Although propolis is generally considered safe, mild adverse reactions, such as urticaria and allergic responses, have been reported, particularly in individuals predisposed to dermatitis [77, 78]. To date, no significant toxic effects associated with propolis use in humans have been consistently reported. However, robust clinical evidence to establish standardized dosage guidelines remains limited. Currently, a daily intake of approximately 70 mg/day (≈1.4 mg/kg/day) has been suggested as a safe level for healthy individuals [77, 78], based on preclinical toxicological data and safety factors applied for extrapolation to human use. Nevertheless, clinical studies have reported the use of doses ranging from 400 to 1500 mg/day [78], with no clear evidence of toxicity in the populations studied. These findings should be interpreted cautiously, as the safety of higher doses has not been sufficiently established, particularly for long‐term use and in individuals with UC.

## 8 Challenges and Prospects

The clinical and industrial applications of propolis face several challenges, particularly due to the high variability in its chemical composition (Figure 5). This variability is strongly influenced by geographic origin, local flora, and environmental conditions, making standardization and reproducibility of its biological effects difficult.

**FIGURE 5:**
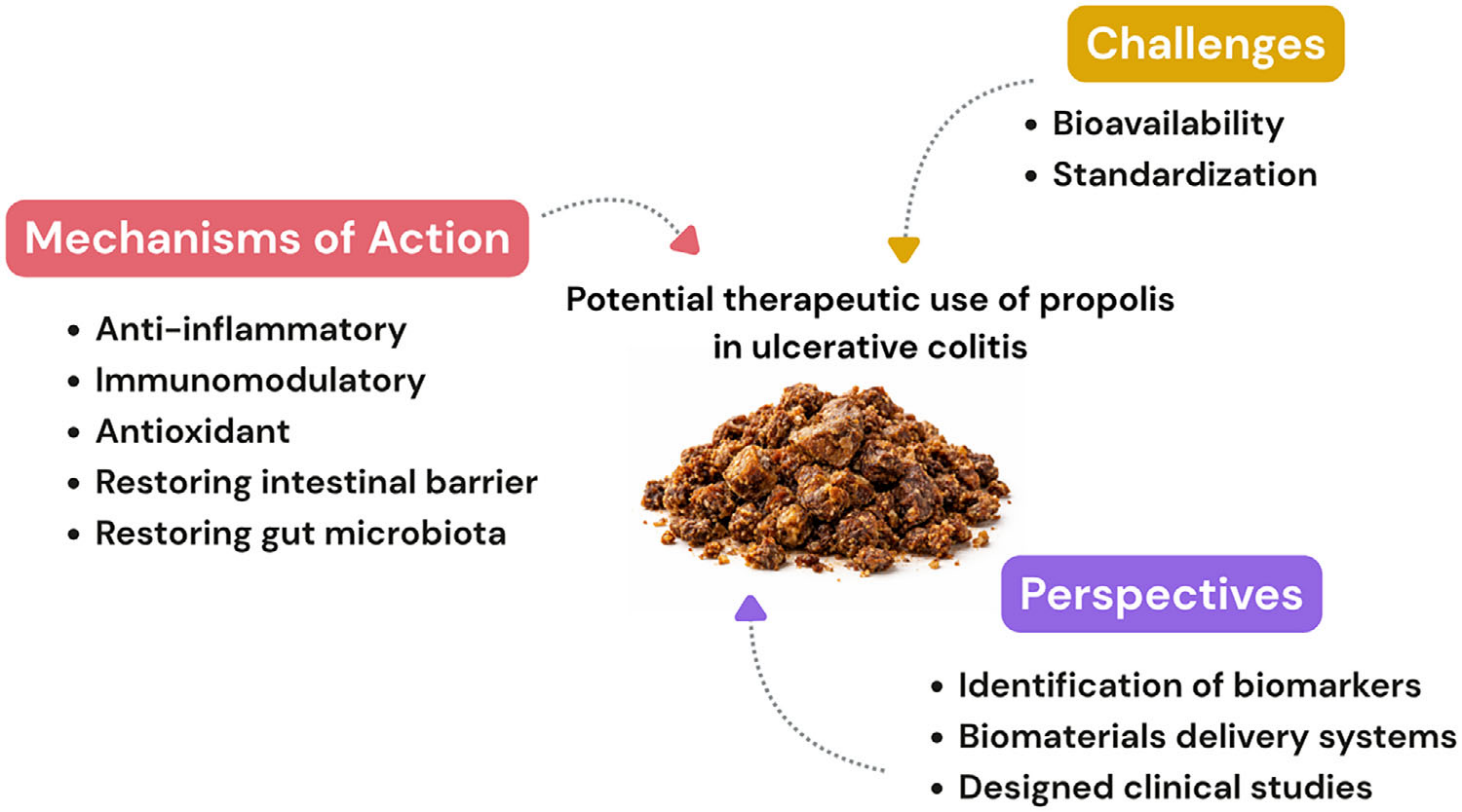
Challenges and prospects related to the therapeutic potential of propolis in the treatment of ulcerative colitis. Created by the authors using Canva.

In this context, analytical strategies based on multiple chemical markers, combined with chemometric analysis, have shown promise for evaluating propolis quality [79]. These approaches enable more robust characterization of propolis samples, facilitating the differentiation of propolis types and the correlation between chemical composition and biological activity. They represent an important tool for addressing the main challenge associated with propolis, namely the variability in its chemical composition.

The use of chemical markers is an important strategy for the standardization and quality control of propolis extracts. Among the compounds employed for this purpose, Artepillin C stands out, particularly as a marker for Brazilian green propolis, as does caffeic acid phenethyl ester (CAPE), the quantification of which has also been proposed in standardization approaches for various types of propolis [80].

Advanced analytical techniques, such as mass spectrometry coupled with separation methods (e.g., liquid or gas chromatography), followed by multivariate chemometric analyses, have proven valuable for the chemical profiling and classification of propolis. These techniques also enable the identification of key biomarkers and bioactive compounds relevant for both therapeutic applications and quality control.

Chemical standardization is a fundamental step not only for the rational therapeutic use of propolis but also for ensuring the reproducibility and reliability of the results obtained in research on its biological properties. According to Bankova [27], the use of standardized material allows specific chemical profiles to be associated with defined biological activities, thereby supporting the development of more precise clinical guidelines. Furthermore, defining the most appropriate extraction methods, formulations, and routes of administration represents another important challenge.

Despite its pharmacological potential, the clinical application of propolis is often limited by its low oral bioavailability. This limitation is attributed mainly to its instability in the gastrointestinal tract, limited transcellular transport across intestinal epithelial cells, and rapid metabolism and excretion, all of which may compromise its effectiveness [40].

Concurrently, significant progress has been made in the development of propolis‐based drug delivery systems. Various nanoformulations—including polymeric nanoparticles, liposomes, nanoemulsions, and hydrogels—have been designed to overcome limitations such as poor aqueous solubility, low stability, and limited bioavailability of propolis‐derived phenolic compounds. These systems show considerable potential for preserving the bioactivity and enhancing therapeutic efficacy.

Other candidates derived from natural products, such as curcumin and resveratrol, also face translational challenges. Despite the anti‐inflammatory effects against IBD observed in preclinical studies, their clinical application is limited by low solubility, stability, and bioavailability. In the case of resveratrol, these obstacles have driven the development of colon‐targeted delivery systems [81], whereas for curcumin, various formulations have been proposed to improve its availability and therapeutic efficacy [82].

## 9 Knowledge Gaps

Although this review sought to evaluate the evidence on the efficacy of propolis and its main bioactive compounds in the treatment of UC, certain limitations must be considered, as they may influence the interpretation and comparability of the results. Significant heterogeneity was observed regarding the doses and routes of administration used in the studies. Although oral administration was predominant, the doses used varied considerably (10 to 600 mg/kg), making it difficult to determine a potentially more effective dose range. Overall, the studies reported beneficial effects across different dosage ranges; however, it was not possible to establish a consistent dose–response relationship, as increasing the dose was not necessarily associated with greater efficacy.

In addition, different experimental models for inducing colitis (DSS, TNBS, acetic acid, and PG‐PS) and different animal strains were used, which limits direct comparisons between studies and the identification of possible differences related to the experimental model or species susceptibility. Treatment and follow‐up periods also varied across studies. Given that UC is a chronic and recurrent disease, the duration of treatment may be a relevant factor in achieving and maintaining a therapeutic response. However, this heterogeneity limits the comparative assessment of the efficacy of propolis extracts and their main bioactive constituents, including their effects on pathways such as NF‐κB/TLR4 inhibition.

It is worth noting that, despite variations in extraction methods and the botanical sources of propolis extracts—factors that can influence their chemical composition—available evidence suggests that these differences do not necessarily preclude comparable biological effects under certain experimental conditions. This observation is supported by studies showing that isolated constituents may also exhibit therapeutic effects when evaluated separately. A study by Wang et al. [33] compared Chinese and Brazilian propolis in rats with DSS‐induced colitis. HPLC‐DAD/Q‐TOF‐MS analysis confirmed that the two products had different polyphenol compositions. Nevertheless, both were administered at the same dose (300 mg/kg) and produced similar effects on various colitis parameters. Several isolated phenolic compounds identified in the Chinese and Brazilian propolis samples, such as apigenin, myricetin, kaempferol, chrysin, and caffeic acid phenethyl ester (CAPE), have already been shown to exert beneficial effects against acute and chronic colitis.

Phytochemical analysis of Egyptian propolis revealed the presence of at least nine flavones, which may also contribute to the antioxidant effects of propolis [38]. Liquiritigenin and formononetin were the compounds found at the highest concentrations in the hydroalcoholic extract of red propolis [43]. Cinnamic acid derivatives present in the ethanolic extract of Brazilian propolis, such as artepillin C, baccharin, drupanin, culifolin, and CAPE, have the potential to suppress inflammatory responses in immune cells [42]. All these observations suggest that the various components of propolis may act cooperatively to produce its protective effects in the treatment of UC.

Thus, although preclinical findings provide relevant evidence regarding the therapeutic potential of propolis, its translation into clinical practice requires further studies, particularly well‐designed clinical trials to establish its efficacy, safety, therapeutic dose, and administration regimen.

## 10 Conclusion

In summary, this review consolidates relevant preclinical evidence regarding the therapeutic potential of propolis and its main bioactive constituents in the context of UC. The beneficial effects observed are primarily associated with its antioxidant, immunomodulatory, and anti‐inflammatory properties (Figure 6).

**FIGURE 6:**
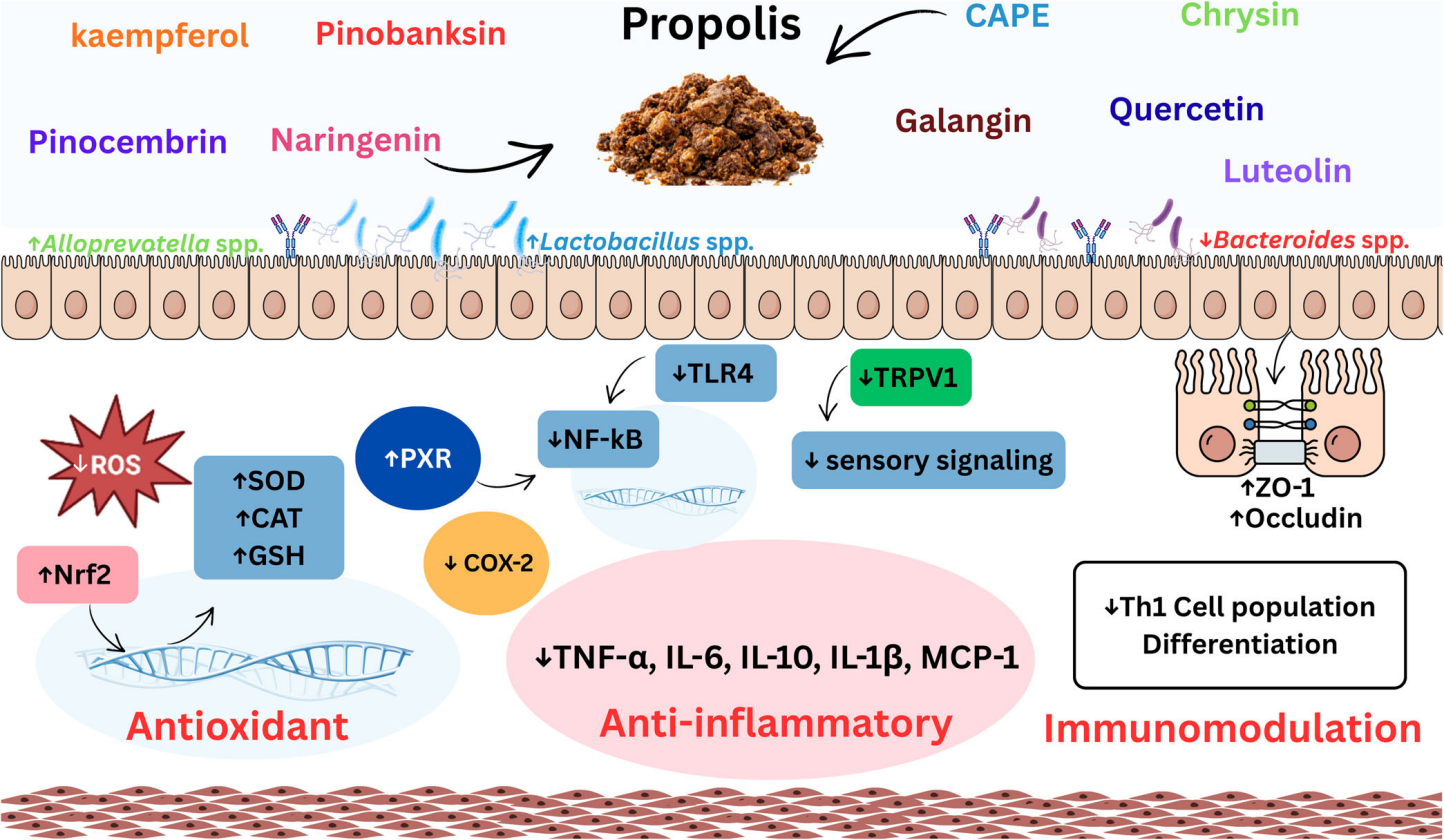
Overview of the cellular and molecular mechanisms underlying the protective effects of propolis and its active compounds in ulcerative colitis. Propolis and its major bioactive constituents (CAPE, pinocembrin, galangin, luteolin, quercetin, naringenin, pinobanksin, chrysin, and kaempferol) attenuate colitis progression through four primary mechanisms: attenuation of inflammatory signaling (suppression of TLR4, NF‐κB, TRPV1, COX‐2, and pro‐inflammatory cytokines/chemokines; activation of PXR; and inhibition of Th1 differentiation); mitigation of oxidative stress (ROS scavenging, Nrf2 activation, and restoration of SOD, CAT, and GSH); enhancement of intestinal barrier function (upregulation of the tight junctions ZO‐1 and occludin); and favorable modulation of gut microbiota composition (*Lactobacillus* spp., *Alloprevotella* spp., and *Bacteroides* spp.). Upward arrows (↑) indicate upregulation; downward arrows (↓) indicate downregulation. Created by the authors using Canva.

Administration of propolis extracts from diverse botanical and geographical origins generally resulted in improvements in clinical and histopathological parameters. These effects include reduced expression of pro‐inflammatory cytokines, decreased infiltration of inflammatory cells into the colonic mucosa, and restoration of epithelial barrier integrity.

At the molecular level, the protective effects of propolis appear to be largely mediated through the modulation of key inflammatory signaling pathways, particularly through the inhibition of NF‐κB activation, resulting in reduced expression of inflammatory mediators such as TNF‐α, IL‐1β, and iNOS. Additionally, propolis has been shown to downregulate TLR4 expression, which may contribute to the attenuation of the exaggerated immune response associated with UC.

Collectively, these findings support the potential of propolis as an adjuvant therapeutic agent for the management of UC. However, extrapolating preclinical findings to the clinical application of propolis requires more robust and consistent evidence regarding its efficacy and safety in humans. This translation depends, above all, on the chemical standardization of extracts and monitoring of specific chemical markers, evaluation of their safety and pharmacokinetic profiles, and conduct of well‐designed randomized controlled clinical trials with rigorous methodological standards.

## Author Contributions

**Thayná Fernandes Barros**: conceptualization, investigation, writing – original draft, methodology, formal analysis, data curation. **Gabriel Gomes Vila Nova**: writing – review and editing, methodology, data curation. **Rosane Nassar Meireles Guerra**: writing – review and editing, formal analysis. **Flavia Raquel Fernandes do Nascimento**: conceptualization, writing – review and editing, formal analysis. **Afonso Gomes Abreu**: conceptualization, writing – review and editing, methodology, validation, formal analysis, project administration, funding acquisition, supervision.

## Funding

This study was supported by the CNPq—Conselho Nacional de Desenvolvimento Científico e Tecnológico (grant numbers: 307035/2022‐0 and Pró‐Amazônia 445615/2024‐9), FAPEMA—Fundação de Amparo à Pesquisa e ao Desenvolvimento Científico e Tecnológico do Maranhão (grant number: Universal 06598/22), and CAPES—Coordenação de Aperfeiçoamento de Pessoal de Nível Superior (FINANCE CODE 001).

## Conflicts of Interest

The authors declare that there are no conflicts of interest.

## Supporting information




**Supporting File**: cbdv71886‐sup‐0001‐SuppMat.docx

## Data Availability

Data sharing is not applicable to this article as no new data were created or analyzed in this study.
