# Supplementary material for: Therapeutic Potential of Propolis in Ulcerative Colitis: A Review of Preclinical Evidence
Source: Chem Biodivers. 2026 Oct 30;23(11):e71886. doi: 10.1002/cbdv.71886 (PMC13720962; doi:10.1002/cbdv.71886)
Supplement: Supplementary file 1 [file CBDV-23-e71886-s001.docx]

**Table S1.** Risk of bias assessment of the included preclinical studies using the SYRCLE tool.

| Study_ID | **Sequence generation** | **Baseline characteristics** | **Allocation concealment** | **Random housing** | **Blinding of experimentalists** | **Random outcome assessment** | **Blinding of outcome assessors** | **Incomplete outcome data** | **Selective outcome reporting** | **Other sources of bias** |
| --- | --- | --- | --- | --- | --- | --- | --- | --- | --- | --- |
| Wang, k. et al. [33] | Low | Low | Unclear | Unclear | Unclear | Unclear | Unclear | Low | Low | Low |
| Wang, J. et al. [34] | Unclear | Low | Unclear | Unclear | Unclear | Low | Unclear | Low | Low | Low |
| Hua, Z. et al. [35] | Low | Low | Unclear | Unclear | Unclear | Unclear | Unclear | Low | Low | Low |
| Alaaeldin, E. et al. [37] | Unclear | Low | Unclear | Low | Unclear | Low | Unclear | Low | Low | Unclear |
| Atta, HA. et al. [38] | Low | Low | Unclear | Unclear | Unclear | Unclear | Unclear | Low | Low | Low |
| Hwang, S. et al. [39] | Unclear | Low | Unclear | Unclear | Unclear | Unclear | Low | Low | Low | Low |
| Franchin, M. et al. [40] | Low | Low | Unclear | Unclear | Unclear | Unclear | Unclear | Low | Low | Low |
| Okamoto, Y. et al. [41] | Unclear | Low | Unclear | Unclear | Unclear | Unclear | Unclear | Low | Low | Low |
| Shimizu, Y; Suzuki, T. [42] | Unclear | Low | Unclear | Unclear | Unclear | Unclear | Low | Low | Low | Low |
| Barbosa Bezerra, G. et al. [43] | Unclear | Low | Unclear | Unclear | Unclear | Low | Low | Low | Low | Low |
| Mariano, LNB. et al. [46] | Unclear | Low | Unclear | Unclear | Unclear | Unclear | Unclear | Low | Low | Low |
| Cury, BJ. et al. [47] | Unclear | Low | Unclear | Unclear | Unclear | Unclear | Low | Low | Low | Low |
| Gonçalves, CC. et al. [48] | Unclear | Low | Unclear | Unclear | Unclear | Unclear | Low | Low | Low | Low |
| Wang, K. et al. [51] | Low | Low | Unclear | Unclear | Unclear | Unclear | Unclear | Low | Low | Low |
| Qian, Z. et al. [31] | Low | Low | Unclear | Unclear | Unclear | Unclear | Unclear | Low | Low | Low |
| Fitzpatrick,LR; Wang, J; Le, T. [54] | Unclear | Low | Unclear | Unclear | Unclear | Unclear | Low | Low | Low | Low |
| Khan, MN. et al. [55] | Unclear | Low | Unclear | Unclear | Unclear | Unclear | Low | Low | Low | Low |
| Wei, X. et al. [56] | Low | Low | Unclear | Low | Unclear | Unclear | Unclear | Low | Low | Low |
| Lai, CS. et al. [57] | Unclear | Low | Unclear | Low | Unclear | Unclear | Low | Low | Low | Low |
| Bi, H. et al. [60] | Unclear | Low | Unclear | Unclear | Unclear | Unclear | Unclear | Low | Low | Low |
| Hu, L. et al. [61] | Unclear | Low | Unclear | Low | Unclear | Unclear | Low | Low | Low | Low |
| Gerges, SH. et al. [62] | Low | Low | Unclear | Low | Unclear | Unclear | Low | Low | Low | Low |
| Comalada, M. et al. [63] | Low | Low | Unclear | Low | Unclear | Unclear | Unclear | Low | Low | Low |
| Guazelli, CF. et al. [64] | Unclear | Low | Unclear | Low | Unclear | Unclear | Unclear | Low | Low | Low |
| Dodda, D. et al. [65] | Unclear | Low | Unclear | Low | Unclear | Unclear | Low | Low | Low | Low |
| Dodda, D. et al. [66] | Low | Low | Unclear | Low | Unclear | Unclear | Low | Low | Low | Low |
| Castangia, I. et al. [67] | Low | Low | Unclear | Low | Unclear | Unclear | Low | Low | Low | Low |
| Park, MY; Ji, GE; Sung, MK. et al. [68] | Low | Low | Unclear | Low | Unclear | Low | Low | Low | Low | Low |
| Li, Y; Shen, L, Luo, H. et al. [69] | Low | Low | Unclear | Low | Unclear | Low | Low | Low | Low | Low |
| Nishitani, Y et al. [70] | Low | Low | Unclear | Low | Unclear | Low | Low | Low | Low | Low |
| Dou, W. et al. [72] | Unclear | Low | Unclear | Unclear | Unclear | Unclear | Low | Low | Low | Low |
| Dou, W. et al. [73] | Low | Low | Unclear | Unclear | Unclear | Unclear | Unclear | Low | Low | Low |
| Al-Rejaie, SS. et al. [74] | Unclear | Low | Unclear | Unclear | Unclear | Unclear | Low | Low | Low | Low |
| Azuma, T. et al. [75] | Unclear | Low | Unclear | Unclear | Unclear | Low | Unclear | Low | Low | Low |

**Note:** Low risk = low risk of bias; Unclear risk = unclear risk of bias due to the absence or insufficiency of information in the article; High risk = high risk of bias. Judgments were based exclusively on information reported in the studies, in accordance with the SYRCLE tool [20].
